# A novel self-gripping long-term resorbable mesh providing temporary support for open primary ventral and incisional hernia

**DOI:** 10.1007/s10856-023-06762-y

**Published:** 2023-11-09

**Authors:** Robert Vestberg, Julie Lecuivre, Amandine Radlovic, Emilie Payet, Yves Bayon, Ludovic Bouré

**Affiliations:** 1 Medtronic – Sofradim Production, Trévoux, F France; 2 Medtronic – North Haven, North Haven, CT USA

## Abstract

A novel synthetic fully long-term resorbable self-gripping mesh has been recently developed to reinforce soft tissue where weakness exists during ventral hernia repair open procedures. This resorbable mesh is a macroporous, knitted, poly-L-lactide, poly-trimethylene carbonate copolymer monofilament mesh with the ProGrip™ technology*, providing grips on one side of the mesh*. A new poly-L-lactide, poly-trimethylene copolymer was developed to provide the required features for mechanical support during at least 20 weeks covering the critical healing period, including resistance to fatigue under cyclic loading conditions, as it occurs in patients. The yarns and mesh initial physical and biomechanical properties were characterized. Then, the mesh mechanical strength was evaluated over time. The mechanical properties of the proposed mesh were found to be above the generally recognized threshold value to mechanically support the repair site of a hernia over a 20-week period during in-vitro cyclic loading test. The mesh performance was evaluated in vivo using a published preclinical porcine model of hernia repair at 4-, 12- and 20-weeks post implantation. The burst strength of the hernia repair sites reinforced with the new mesh were higher at 4 & 12 weeks and comparable at 20 weeks to the one of the native abdominal walls. At all time points, the mesh was well tolerated with moderate inflammation and was fast integrated in the abdominal wall at 4 weeks. Particularly, the grips were nicely engulfed in the newly formed connective tissue. They must facilitate the anchoring of the mesh by their extension from the mesh and their mushroom shape. The preclinical data of the self-gripping resorbable mesh suggests that it has all the favorable characteristics for future clinical use during ventral hernia repair open procedures.

**Graphical Abstract:**

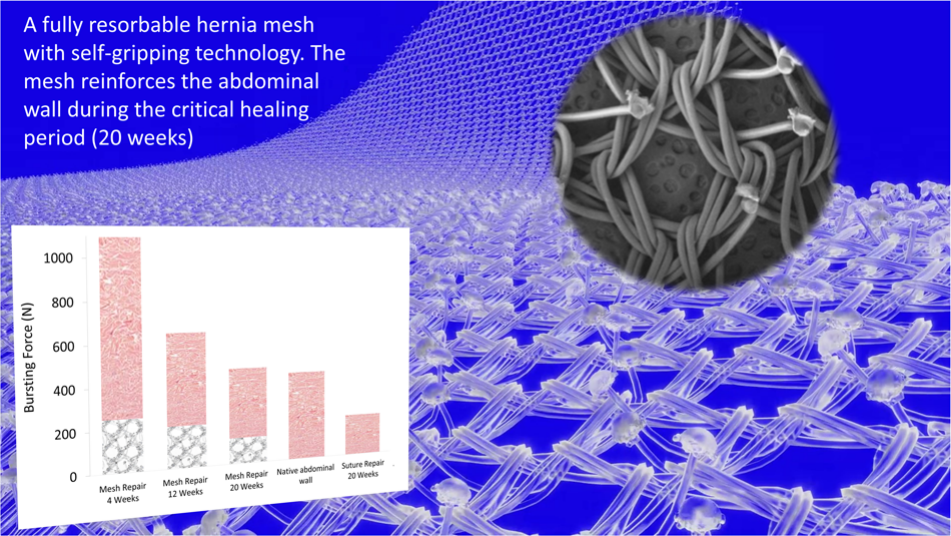

## Introduction

Poly(glycolic) acid was originally reported in 1954 and approved by the FDA in 1969 as the first biodegradable synthetic polymer to be used to manufacture surgical suture material [1]. Since then, synthetic biodegradable polymers have been extensively reported in the literature as materials of choice in the conception of implants for soft and hard tissue repair. They include, as most used, polylactic acid (PLA), polyglycolic acid (PGA), poly(ε-caprolactone) (PCL), poly-hydroxybutyrate (PHB) and their copolymers. These polymers can be formulated and processed in multiple ways to accommodate the most desired design of the implants, whether they are surgical sutures, bone orthopaedic plates, screws, tacks, stents or woven or knitted textiles. With well-understood degradation mechanisms, the monomer ratio, molar mass, and crystallinity of these copolymers can be adjusted to combine mechanical properties and degradation time that fit the specifications of the clinical needs [2]. A large range of controlled degradation times is accessible from several weeks to years, as illustrated in absorbable surgical sutures. For example, poly-lactide-co-glycolide (PLGA) sutures with different ratios of lactic acid to glycolic acid are reported to be degraded from 40 to 60 days to more than 1.5 years. With a long history of safe use and biocompatibility profiles, biodegradable synthetic polymers are commonly well accepted by medical device regulatory bodies for clinical use [3]. Moreover, their manufacturing processes are well defined and mastered and support repeatability and reproducibility requirements and cost-effectiveness [4, 5].

Recently, synthetic biodegradable polymers gained attention for abdominal hernia repair, particularly for primary and incisional ventral hernia indications [6–11]. They present an alternative to permanent polymers (eg. polyethylene terephthalate [PET], polypropylene [PP]) and materials of biologic origin (eg. porcine dermis xenografts). Their use has also the objectives to reduce possible long-term complications such as chronic inflammation, chronic pain, and infection thanks to material elimination and to promote tissue regeneration [6–11]. Synthetic biodegradable polymers for hernia repair meshes should be selected to support mid/long term degradation with expected mechanical reinforcement of the abdominal wall during the wound healing phase. If the choice of the polymer is important to guarantee the performance of the implants, the specific design of the mesh is also critical to offer adequate mechanical properties over time and appropriate porosity to allow tissue ingrowth through the mesh and its remodelling. Indeed, the reinforcement of abdominal wall tissues by meshes is affected by a number of critical parameters including the mechanical properties (e.g. tensile strength, elasticity), the pore size, the surface density, the chemical composition [12–14]. The handling the mesh is also a factor to consider even if it does not necessarily have a direct impact on clinical outcomes.

Currently approved slowly degradable devices for hernia repair are i) GORE® BIO-A® and GORE® ENFORM® (Gore) consisting of a non-woven mesh made of poly(glycolide:trimethylene carbonate) copolymer, ii) DuraSorb® Monofilament Mesh (SIA Health), a knitted mesh made of polydioxanone fibers, iii) Phasix™ mesh (BD), a monofilament knitted mesh composed of poly-4-hydroxybutyrate fibers and iv) TIGR® Matrix Surgical Mesh (Novus Scientific), a multifilament knitted mesh, consisting of two types of fibers: a fast-resorbing fiber (40% of weight, copolymer of glycolic acid, lactic acid and trimethylene carbonbate) and a slow-resorbing fiber (60% of weight, copolymer of lactic acid and trimethylene carbonate). They are intended to be used in abdominal wall repair where weakness exists to reinforce soft tissues. Limitations are more or less disclosed in the Instructions For Use (IFU). For example, most of the commercially approved resorbable meshes should not be used in repairs where permanent wound or organ support from the mesh is required. The use of these meshes has been supported by preclinical animal studies and clinical studies (GORE® BIO-A®, TIGR® Matrix Surgical Mesh and Phasix™ mesh) in different settings. All meshes mentioned above are very clearly distinguished from partially degradable devices such as the meshes of the ULTRAPRO® range (Ethicon) providing sufficient mechanical properties expected for the approved indications of hernia repair, but reducing the mass remaining permanently, after the disappearance of the resorbable part. They are also very different from the ProGrip range of meshes (Medtronic), made of knitted PP or PET with polylactic acid monofilament grips that aid in the positioning and fixation of the meshes for approved hernia repair indications.

Herein we report the development and the characterization of a new mesh by including a proprietary biodegradable copolymer of lactic acid and trimethylene carbonate, designed to offer an optimal mechanical fatigue resistance profile for the reinforcement of soft tissue where weakness exists during open ventral hernia repairs. This new absorbable mesh also offers micro-grips (ProGrip™ technology) on one side, facilitating the positioning of the mesh during surgery and help to maintain the mesh in contact with the tissue supporting an excellent tissue integration. Microgrips are an innovative feature, not supplied equivalently, in all existing commercial meshes, which come in the form of flat sheets. The mesh has been extensively evaluated and characterized during in vitro tests and animal studies in abdominal wall repair models.

## Materials & Methods

### Materials

The new resorbable mesh is made using warp knitting technology. It is composed of monofilament fibers obtained by extrusion of poly(L-lactide) trimethylene carbonate (PLLA-TMC) manufactured by Medtronic plc, North Haven, USA. The poly(L-lactide) trimethylene carbonate copolymer (PLLA/TMC, 80/20 mol/mol) was designed as a triblock copolymer with the A-B-A’ structure wherein the A and A’ blocks each include polylactide, the B block includes poly-trimethylene carbonate and polylactide manufactured according to Patent US11447602B2, Walter et al., 2018. The textile-based meshes used in this study were manufactured by Medtronic plc, Sofradim Production, Trévoux, France using proprietary pattern and process and sterilized with ethylene oxide.

Progrip™ Self-Gripping Polyester Mesh and Parietene™ Flat Sheet Mesh were used as reference products and were sourced from Medtronic plc, Sofradim Production, Trévoux, France.

### Methods

#### In vitro degradation

The mesh in vitro degradation was characterized according to the ISO 13781:2017 Standard “Implants for surgery Homopolymers, copolymers and blends on poly(lactide) — In vitro degradation testing”. Degradation was performed in a 1/15 molar phosphate buffer solution (Sorensen buffer), pH = 7.4 at 37 °C containing 0.01% of sodium azide to assure sterility throughout the testing. Approximately 1.5 g of sterile mesh was used put in tubes containing 50 ml of buffer solution (Sterile Falcon 50 ml - BD352098). The samples were incubated at 37 °C and the pH was monitored at least every 6 weeks. When the pH dropped below 7.2, the buffer solution was changed. After degradation, the buffer was decanted and aspirated, and the mesh sample was rinsed with distilled water. If needed, the tubes were centrifuged before removing the liquid (highly degraded samples).

#### Gravimetric mass

Gravimetric mass was measured during degradation time. Mesh samples (*n* = 5 per time-point) were dried in a vacuum desiccator until constant weight was obtained ( ± 1% of sample weight). The dried samples were weighed, and the final mass was compared to the initial mass to calculate the gravimetrical mass loss.

#### Molar mass (Mw, Mn and Polydispersity Index)

Absolute molar mass measurements were performed during degradation time using a chromatography system Waters APC (Advanced polymer chromatography system, Waters Corporation,) with light scattering and refraction index (RI) detection. The operating temperature was 40 °C. The HFIP solvent (1,1,1,3,3,3-hexafluoro-2-propanol) used was purchased from Fisher Scientific. Analyses were performed with an injection volume of 30 µL, a flow rate of 0.3 mL/min and a sample concentration of 1.5 mg/mL. Agilent Technologies columns PL HFIP gel Guard 50*4.6 mm and two Agilent Technologies columns PL HFIP gel 250*4.6 mm were used. The RI (Optilab T-rEX) detector and the multiangle light scattering detector Dawn® Heleos™ II (wavelength of 660.4 nm) used were produced by Wyatt technologies. The light scattering measurements and the RI measurements were performed by using Astra 6.1.7 software (Wyatt technologies).

#### Differential Scanning Calorimetry (DSC)

DSC measurements were carried out on a Mettler Toledo DSC thermal analyzer, integrated with software STARe. For each reference, five mesh samples (5–6 mg) were submitted to a heating scan of 200 °C (10 °C/min), a cooling scan of 0 °C (10 °C/min) and a second heating scan of 200 °C (10 °C/min). Glass transition temperature (Tg), melting temperature (Tm) and degree of crystallinity (Xc) were determined from the second heating ramp. A reference enthalpy of melting of 93.1 J/g was used to calculate the crystallinity of PLA [15].

#### Proton Nuclear Magnetic Resonance (^1^H NMR) spectroscopy

Copolymer composition and monomer residues were determined using ^1^H NMR (300 MHz) spectroscopy on a Bruker Fourier 300 at 30 °C using CDCl_3_ as solvent.

#### Bursting strength

Bursting strength measurements were performed before and during degradation, according to the ASTM 6797-15 Standard “ Test Method for Bursting Strength of Fabrics Constant-Rate-of-Extension (CRE) Ball Burst Test” using a traction/compression testing machine – Tinius Olsen model H5KS (Tinius Olsen. Test specimen dimensions of 65 mm x 65 mm were used with a testing area of a diameter (Ø) of 44.45 mm. A ball of Ø25.4 mm was used with a crosshead speed of 305 mm/min and a pre-load of 0.1 N.

#### Uniaxial Tensile strength

Uniaxial tensile strength measurements – before and during degradation – were performed according to a strip method using a traction testing machine – Tinius Olsen model H5KS (Tinius Olsenand using following test conditions: test specimen dimensions of 25 mm × 60 mm were used with 40 mm between the jaws and a crosshead speed of 20 mm/min with a pre-load of 0.5 N.

#### Suture pull-out strength

Suture pull-out strength measurements – before and during degradation – were performed using a traction testing machine – Tinius Olsen model H5KS (Tinius Olsen) and using following test conditions: test specimen dimensions of 25 mm × 50 mm were used. A USP2 suture was passed medially at 10 mm from the edge of the test specimen, through a pore. The assembled specimen was positioned on the traction testing machine with 40 mm between the jaws, so that the suture part of the specimen was clamped in a thread clamp, while the mesh part of the specimen was clamped in a flat clamp with about 20 mm of mesh in-between the clamps. A crosshead speed of 20 mm/min was used.

#### Tear strength

Uniaxial tensile strength measurements – before and during degradation – were performed according to a trouser-shaped specimen method using a traction testing machine – Tinius Olsen model H5KS (Tinius Olsen,)and using following test conditions: test specimen dimensions of 50 mm × 90 mm with 60 mm slit length were used with 30 mm between the jaws and a crosshead speed of 100 mm/min.

#### Samples for mechanical testing under cyclic mechanical loading

The mesh samples were cut to a circle of Ø29 cm and clamped in the testing module including a hermetic silicone membrane. The mesh were placed towards the buffer solution (phosphate buffer) with the grips facing away from the membrane. Cycling loading was applied to the mesh by pneumatically loading the hermetic silicone membrane. The effective testing area was a circle of Ø23 cm.

Cyclic loading was applied to the sample simulating human breathing cycles using a sinusoidal pressure cycle of 26 mmHg applied at a frequency of 0.25 Hz. The biomechanical effects of physical activity were simulated 8 times per day by 5 consecutive Valsalva-like square pressure cycles of 52 mmHg with 3 seconds hold time. The pressure applied to each cycle in this study was selected from the mean intraabdominal pressure data reported by Cobb et al. [16], notably during standing (20 mHg) and Valsalva maneuver - coughing and straining against a closed epiglottis during standing - (39.7 mmHg). The pressures to be applied on samples were calculated in such a way: i) by considering the difference in sample effective testing area and the area of the abdominal wall, ii) to get equivalent force onto samples vs abdominal wall.

At the end of a degradation period, samples were dried and cut to prepare test specimens. Test specimen for ball burst (65 mm × 65 mm) was extracted from the center part of the mesh sample.

Before mechanical testing the testing specimens used for ball burst and tensile testing were conditioned in water for 60 min ± 15 min at 37 °C.

#### Porcine preclinical studies

The in vivo performance of the new mesh was documented during preclinical studies. These studies were conducted following the Directive 2010/63/EU of the European Parliament and of the Council of 22 September 2010 on the protection of animals used for scientific purposes (OJEU of 20-10-2010). All studies were also compliant to Good Laboratory Practice (GLP). They have been reviewed and approved by the Ethical Committee.

#### Evaluation of the soft tissue reinforcement provided by the new mesh at 4-, 12- and 20-weeks following implantation

Forty-one (*n* = 41) intact male Yucatan miniature swine (INRA, Rennes, France) weighing between 41 and 63.5 kg at the time of mesh implantation were included in the study with 4, 12, and 20 weeks timepoints to evaluate the functional performance of the repair site with the new resorbable mesh compared with Native Abdominal Wall (NAW).

Ten (*n* = 10) Yucatan miniature swine were dedicated to evaluating the New Resorbable Mesh local tolerance and material degradation as per ISO 10993-6 (2016), 4-, 12- and 20-weeks following implantation. Implant-site tissue samples were trimmed, paraffin-embedded, and microtome sectioned to 4 μm and stained with hematoxylin and eosin (H&E) and Masson’s Trichrome (MT) for assessment via light microscopy. Qualitative and semi-quantitative histological evaluation of each implanted site was performed by a board pathologist. The scale used was 0: absent, 1: slight, 2: moderate, 3: marked, 4: severe.

##### Surgical technique and post-surgery care

Yucatan miniature swine were premedicated with an intramuscular (IM) injection of 0.2 mg/kg of diazepam (Valium®, Roche Farma S.A.), and 20 mg/kg of ketamine (Ketamidor®, Richter Pharma). Anesthesia was induced by an intravenous injection of propofol (Propofol®, Sandoz), and maintained by inhalation of O_2_-Sevoflurane (1–4% mixture, SEVOTEK®, Karizzo). After the ventral abdomen preparation for aseptic surgery, a midline laparotomy of 20–30 cm line was performed leaving the peritoneum intact. After pre-peritoneal dissection a 3 cm-diameter full-thickness abdominal wall defect that included the muscle and fascia layers was created on each side of the midline, centered 4–6 cm laterally from the umbilicus. The defects were repaired with USP0 absorbable suture material and reinforced with one 9 cm diameter circle of the new resorbable mesh fixated with 14 fixation devices (AbsorbaTack™, Medtronic plc, North Haven, USA). Once completed, the abdominal midline was closed using standard closure techniques. The animals were recovered and individually housed in pens during the study duration. The abdominal region was inspected daily to assess the condition of both the wound line and subcutaneous tissue (seromas and/or hematomas) during the postoperative period.

The pigs were humanely euthanized with a protocol compliant to Directive 2010/63/EU. A necropsy consisting of examination of the implant sites was performed by a board-certified veterinary pathologist on all animals to determine the presence of seroma, herniation or any other side effects and to macroscopically characterize the mesh tissue integration. Abdominal wall was excised, the peritoneum and the associated fat were removed. The skin, subcutaneous tissue and the cutaneous muscle were dissected to expose the surgical sites. In addition, for each animal, Native Abdominal Wall (NAW) was harvested caudal to the surgical sites for biomechanical testing to obtain a reference value of the NAW biomechanical behavior.

##### In-vivo bursting strength

The explanted test and NAW specimens were subjected to burst testing using a traction/compression testing machine – Instron 5965 Series universal testing system (Instron, Norwood, USA). Repaired sites were sampled centering the defect created during surgery in 10 by 10 cm test specimens with a testing area Ø44.45 mm. A ball of Ø25.4 mm was used with a crosshead speed of 305 mm/min and a pre-load of 0.1 N.

#### Evaluation of the new resorbable mesh fixation strength during the tissue integration process

Seven (*n* = 7) female white pig weighing 49–57.5 kg at the time of implantation surgery were included to evaluate the fixation strength of the new resorbable mesh compared to the one of Progrip™ Self-Gripping Polyester Mesh 8 weeks following implantation. Parietene™ Flat Sheet Mesh was also included considered as a negative control.

##### Surgical technique and post-surgery care

Pigs were prepared for surgery and premedicated with an intramuscular (IM) injection of 0.2 mg/kg of diazepam (Valium®, Roche Farma S.A.), and 20 mg/kg of ketamine (Ketamidor®, Richter Pharma).

Anesthesia was induced by an intravenous injection of propofol (Propofol®, Sandoz), and maintained by inhalation of O_2_-isoflurane (1–4% mixture, Isoflo®, Zoetis Spain). After the ventral abdomen preparation for aseptic surgery, a midline laparotomy of 20–30 cm line centered at the umbilicus was performed leaving the peritoneum intact. A pre-peritoneal dissection was performed to lift the abdominal walls on both sides on the incision. The test and control articles with a size of 5 cm × 10 cm (width x length) were applied 1–2 cm laterally to the linea alba on both sides against the rectus posterior fascia of the abdominal wall. For the articles with grips, the side with grips was placed directly against the posterior fascia of the rectus abdominis muscle. All articles were fixated with 4 stitches (one in each corner) of USP3-0 absorbable suture. Each animal received 4 randomly assigned meshes in the preperitoneal space. The abdominal midline was repaired with standard closure techniques.

##### Mechanical testing using a peeling test

The pigs were humanely euthanized with a protocol compliant to Directive 2010/63/EU. Following euthanasia, abdominal wall was excised, the peritoneum and the associated fat were removed. The skin, subcutaneous tissue and the cutaneous muscle were dissected to expose the surgical sites. Samples were harvested with the muscle by trimming the samples around the meshes. Then, the mesh was dissected from the muscle to its whole width and over a length of 2 cm. A T-peel test was carried out by using a traction/compression testing machine Instron 5965 Series universal testing system (Instron, Norwood, USA), under the following conditions: speed of extension or strain rate: 50 mm/min, and pre-load: 0 N.

The parameters recorded were the maximum force (N), the work (J) and the peel length (mm). The peel work (mJ) which consists in the area under the curve, and the ratio of peel work over total peel length (mJ/mm) were calculated for each test samples corresponding to the mean peel strength (N).

#### Statistical analyses

Minitab software (version 18.0, Minitab, Inc., PA) was utilized to perform all statistical analyses. Two-sample t-test were performed and for data in which three or more groups of data were compared, one-way analysis of variance (ANOVA) were performed followed by a Fisher’s LSD post-test as appropriate.

## Results & discussion

### Fiber & mesh properties

#### Fiber characterization

The physicochemical properties of the fibers made of three distinct PLLA-TMC polymer batches are reported in the Table 1. They are rather consistent from batch to batch, within a narrow range (less than ± 10%), except of Mn and the PDI. The composition of the polymers agrees with the L-lactide: trimethylene carbonate weight ratio target, 80:20, w/w as checked by NMR and reported in the Table 2. The residual monomers were analyzed before the cleaning process of the fibers by supercritical CO_2_ technology, deemed to further decrease the content of monomers, soluble in supercritical CO_2_ [17]. Monomer residues of polylactic acid or derivatives can accelerate the hydrolytic degradation of the polymers at concentrations above 5% by weight [18–20]. After the cleaning process, monomer residues on the finished new resorbable mesh were observed in the 0.5–1.0% range (data not shown). The melting temperature (Tm) and glass transition temperature (Tg) are in the range commonly observed for copolymers of lactic acid (LA) and trimethyl carbonate (TMC), about 170 °C and 50 °C for Tm and Tg, respectively [21–23]. As expected, the Tm and Tg are much reduced when compared to the values of PLLA (Tm, 196 °C and Tg, 68 °C as reported by Fuoco et al. [23]). The TMC- component of PLLA-TMC should lead to more suitable polymers for implants - i.e., more elastic - than PLLA with higher Tg and crystallinity. Slight changes of the polymerization conditions can lead to substantial molar mass (Mw) differences. The values of the copolymer of this study were bracketed between the low and high Mw values (56 – 300+ kDa) of the copolymers synthetized by Fuoco et al. [23]. This team has also reported that the thermal properties of the copolymers of LA & TMC as well as the crystallinity are influenced not only by the catalyst used for the polymerization, but also by the Mw.

**Table 1:** Molar mass & DSC analysis of the PLLA-TMC yarns

Parameters	Batch #1	Batch #2	Batch #3
Mw (kDa)	137 ± 6	122 ± 3	118 ± 2
Mn	104 ± 5	66 ± 10	59 ± 14
PDI	1.3 ± 0.1	1.8 ± 0.3	2.1 ± 0.5
Tm (°C)	174.9 ± 1.0	171.3 ± 2.1	169.0 ± 1.4
Tg (°C)	54.8 ± 0.7	55.1 ± 1.0	53.7 ± 0.3
Crystallinity (%)	43.0 ± 2.3	40.7 ± 1.8	40.0 ± 1.5

**Table 2:** ^1^H NMR analysis of three batches of PLLA-TMC yarns

Parameters	Batch #1	Batch #2	Batch #3
% L-lactide	78.3	78.2	78.1
% TMC	21.7	21.8	21.9
% Total residual monomers	5.4 ± 0.1	5.5 ± 0.1	5.8 ± 0.1

#### Mesh features

The new resorbable mesh shows similar mechanical behavior in warp and weft directions as reported in the Table 3. The pore size is considered as large, i.e., over 1 mm. Porosity is one first critical parameter which can be easily adjusted by knitting processes. It drives the tissue integration of the mesh in abdominal wall over the time and the extent of the foreign body reaction and granuloma formation [13, 24]. The new resorbable mesh displays values in the large range, with porosity ranging between 1 and 2 mm. The thickness and the surface density are in the high range since the mesh includes grips on one of its sides. This new mesh can be classified as an extra-thick mesh according to Deeken et al. [12]. This is due to the grips adding an extra-layer to the mesh providing fixation capacities to the surrounding tissues.

**Table 3:** Mechanical properties of the new resorbable mesh vs TIGR® Matrix Surgical Mesh

Test	New Resorbable Mesh		TIGR® Matrix Surgical Mesh	
Pore size (mm) (Width x height)	1.4 ± 0.1 × 1.4 ± 0.1 (larger pore) 1.2 ± 0.1 × 1.4 ± 0.1 (smaller pore)		1.0 ± 0.0 × 1.0 ± 0.0 (larger pore) 1.0 ± 0.1 × 0.9 ± 0.0 (smaller pore)	
Thickness (mm)	1.7 ± 0.1		0.9 ± 0.1	
Surface density (g/m²)	171 ± 2		148 ± 3	
Ball burst (Ø25.4 mm) maximum force (*N*)	464 ± 19		449 ± 27	
Ball burst (Ø25.4 mm) tensile strength (N/cm)	108 ± 5		105 ± 4	
	Warp	Weft	Warp	Weft
Uniaxial tensile maximum force (*N*)	119 ± 12	118 ± 23	164 ± 18	163 ± 16
Uniaxial tensile elongation at maximum force (%)	52 ± 4	62 ± 7	50 ± 7	49 ± 4
Tear strength (*N*)	44 ± 4	37 ± 3	32 ± 2	36 ± 4
Suture pull-out strength (*N*)	53 ± 4	51 ± 5	44 ± 5	57 ± 4

The mechanical properties of the new resorbable mesh have been extensively analyzed to guarantee the use of the mesh for its intended functions, notably as a temporary mechanical reinforcement during the critical wound healing period, according to the experience of the authors of this study.

It is hard however to compare head-to-head these values with the literature due to the lack of consensus on the mechanical tests and of details on their exact conditions (e.g., size of textile samples, setting of tensile/ball burst machines, strength units and/or conversion in N/cm).

The ball burst test result may be used after the corresponding tensile strength (in N/cm) is calculated. [12]. The tensile strength is calculated based on the measured maximum force and deflection at maximum force, as well as the geometry of the test setting (ball diameter, testing area diameter).

A series of commercial hernia meshes – amongst the most used in the clinics – returned values above 50 N/cm for a specification set at 24 N/cm (for repair without bridging), in the ball burst test, value indicated as a threshold value to mechanically support the intended functions of the tested meshes. The new resorbable mesh is tested at a value slightly above 100 N/cm, thus an acceptable value. The mechanical properties of the New Resorbable Mesh were compared to the ones of TIGR® Matrix Surgical Mesh made of knitted multifilaments of similar chemistry (see the Introduction part), in the same testing conditions, head to head. Notably the Ball Burst test returned similar results. The tear strength and the suture pull-out strength test results are also in the same range. The maximum strength in the uniaxial test is higher for TIGR® Matrix Surgical Mesh, but the values for both meshes are acceptable [12].

### Degradation profile after cyclic mechanical loading (see Table 4)

As described in the Materials & Methods section, an in vitro assay was developed to assess the mesh degradation profile, under conditions mimicking clinical parameters (i.e. clinically relevant cyclic loading patterns, 37 °C). At all times tested, no breaking point was observed in the meshes. But the new resorbable mesh gradually lost its mechanical properties over time, with a loss of approximately 80% of Ball burst maximum force value over 26 weeks. The molar mass decreases by about 50% at the same time. The tensile strength in the ball burst test is consistently above the threshold of 24 N/cm [12]. LA and TMC copolymers were originally designed to improve the flexibility of PLLA, by reducing crystallinity and Tg values. The reduction of Tg is indeed known to generate less plastic materials [22]. It is known that dynamic loading accelerates the degradation of PLLA compared to static loading [25, 26]. The degradation profile of the new resorbable mesh suggests the mesh has the required mechanical strength, during at least the first 20 weeks, after implantation.

**Table 4:** Mechanical and molar mass results for the degradation of resorbable mesh with mechanical solicitation

Test		Degradation Time (Weeks)						
		0	1	3	6	13	20	26
Ball Burst Ø25.4 mm	Force Max (*N*)	464 ± 19	376 ± 28	261 ± 16	235 ± 15	198 ± 12	126 ± 13	86 ± 20
	Tensile strength (N/cm)	108 ± 5	96 ± 4	89 ± 4	94 ± 5	95 ± 4	72 ± 2	52 ± 13
Molar mass,	M_w_ (kg/mol)	132 ± 6	120 ± 8	106 ± 10	104 ± 10	84 ± 6	78 ± 3	63 ± 2

### In vivo studies in pigs

#### Evaluation of the soft tissue reinforcement provided by the new mesh at 4-, 12- and 20-weeks following implantation

The strength of mesh-tissue repair was measured at three different timepoints. The first timepoint was selected during the initial period of healing (4 weeks), the second during the tissue remodeling phase of the repair (12 weeks) and the last one on mature tissues (20 weeks).

##### Survival, Clinical observations

One (1) animal out the 51 implanted was euthanized before the end of the study and was included in the histologic group 12 weeks following implantation. This animal presented a weakness in hind legs with difficulties in standing up. A reduced feed consumption was observed with a loss of more than 20% of its initial weight. A complete necropsy report was performed by a board-certified pathologist, concluding that the implantation sites had not been identified as the cause. Others, superficial subcutaneous infections of the skin in four Yucatan (20 weeks group), were documented and resolved with a prolonged period of wound cleaning from 7 days to 14 days after the surgery and antibiotic treatment. An additional surgery was performed two weeks after the initial surgery on one animal (group 20 weeks) to repair a failure of 15 cm of the laparotomy closure (on the midline). In the 4 weeks group, one animal presented a swelling and stiffness in the anterior member. Meloxydyl ® (CEVA Sante Animale, France, intramuscular) and buprenorphine (Bupaq, Richter Pharma), Cefadroxil (20 mg/kg/day PO, Cefacure MSB Animal Health, Salamanca) was administered until the end of the study. In the 12 weeks group one animal presented 14 days after the surgery an abscess in a caudal part of the skin incision treated with Cefradroxyl (20 mg/kg/day PO, Cefacure MSB Animal Health, Salamanca) and Blastoestimulina 10 mg/g (Industrias Farmacéuticas Almirall, S.A., Barcelona, Spain) for 10 days.

##### Macroscopic observations

All implants were incorporated into host tissue at all time points. The defects were healed and the meshes were visible. No serous fluid collections, hematomas, herniations, mesh migrations, or areas of tissue necrosis were noted at necropsy.

##### Mechanical testing of explants

**Fig. 1:**
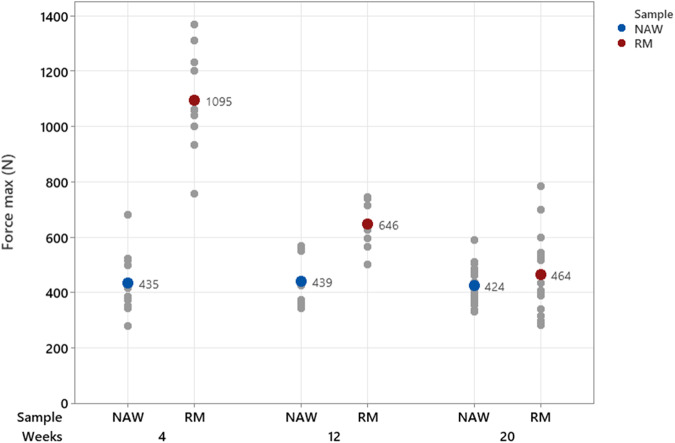
Bursting strengths of explants at 4, 12, and 20 weeks, after implantation (individual values and mean). NAW Native Abdominal Wall, RM New Resorbable Mesh

Biomechanical testing was performed on the new resorbable mesh repair sites as well as on native abdominal tissue (NAW)—at 4, 12, and 20 weeks. A summary of the data is presented in Fig. 1. The NAW burst maximum force was stable, as shown by the mean measurements at 4 weeks (430 N ± 101), 12 weeks (424 N ± 87), and 20 weeks (424 N ± 67). The maximum force of the new resorbable mesh repaired sites were significantly higher to the maximum force of the NAW at 4 weeks and 12 weeks (*p* < 0.001) and equivalent at 20 weeks.

The observed level of performance of the new resorbable mesh is in accordance with the in vitro mechanical fatigue test results. In the animal study, as the mesh degrades, so does the strength of the repair, provided by the mesh itself. This was similarly observed in the cyclic mechanical loading test. The mesh has fulfilled its function as a mechanical reinforcement during the critical wound healing period, at all implantation times tested, up to 20 weeks. It both supports the repair of abdominal wall with equivalent mechanical properties to the native tissue and the formation of a healing tissue in the porcine performance model. The ball burst maximum force of the tested sites with the mesh were observed above the strength of the native abdominal wall, at the intermediate time points at 4 & 10 weeks. This is explained by the mechanical contribution of the mesh. Similar behaviors of hernia mesh have been reported in porcine models [27]. At 20 weeks, the repaired abdominal wall started to take over the new resorbable mesh in terms of mechanical properties. This is exactly the expected scenario for the new resorbable mesh which has been designed as a temporary support of the healing of abdominal wall defects.

Similar reports on the behavior of resorbable meshes have been reviewed in animal models of hernia [11]. Abdominal wall defects have been completely repaired by GORE® BIO-A® mesh (Gore), TIGR® Matrix Surgical mesh (Novus Scientific) and Phasix™ mesh (Bard) with no recurrences during the time of the animal studies. As suggested by Miserez et al. (2019), the promising experimental studies should be confirmed by randomized controlled trials and prospective registries in humans with a sufficiently long follow-up period, to reveal the potential advantages in clinical practice. First series of published clinical studies indicates the potential of TIGR® Matrix Surgical mesh and Phasix™ mesh for preventing and/or repairing ventral hernias [28–30].

**Fig. 2:**
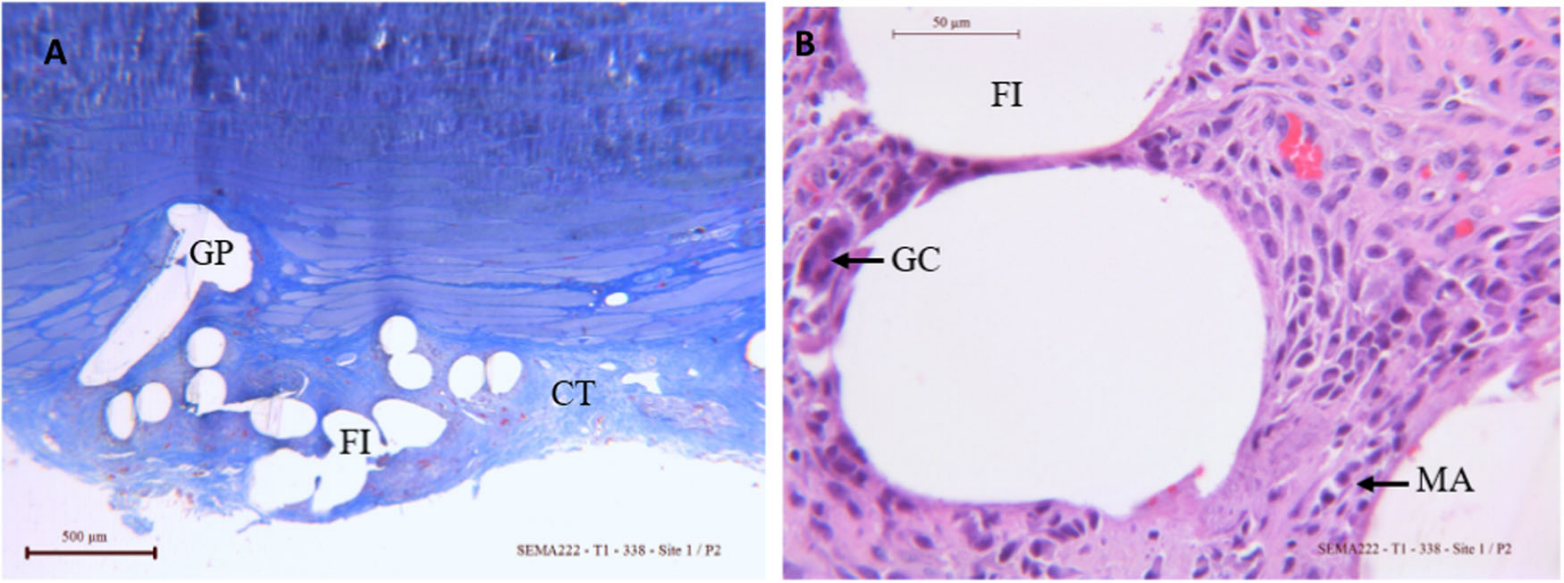
Representative photomicrographs of the new resorbable mesh implanted at 4 weeks A: **A** 2.5X view of stained mesh with Masson Trichrome. **B** A 25x view of stained mesh fibers with Hematoxylin Eosin FI Fiber, GC Giant cell, GP grip, MA Macrophages

Histologic analyses semi quantitative measurement showed the maximum score of 4 meaning a complete tissue ingrowth and full tissue integration of the new resorbable mesh 4 weeks after implantation and up to 20 weeks with no adverse local tissue reaction observed. The grips of the new resorbable mesh were visible and were fully integrated 4 weeks after implantation. The Fig. 2A shows the formation of a well-organized connective tissue around the mesh and the grips.

**Fig. 3:**
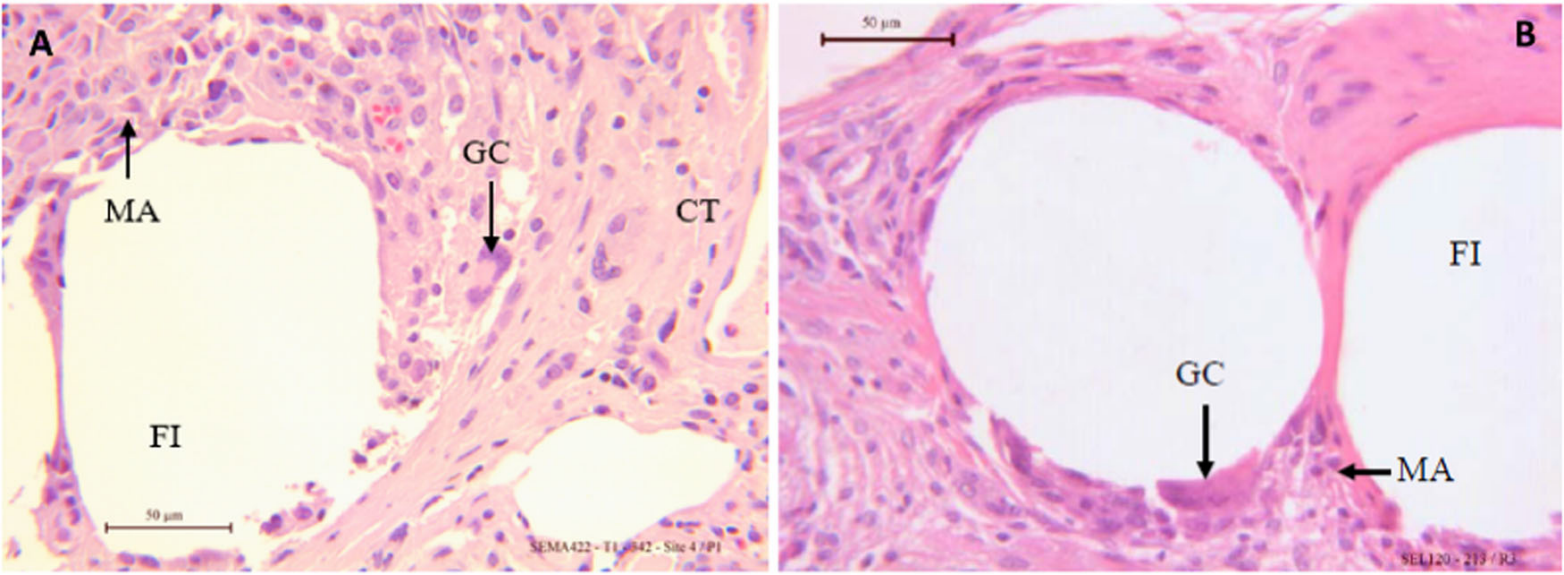
Representative photomicrographs of the new resorbable mesh implanted at 12 weeks (**A**) and 20 weeks (**B**) with Hematoxylin Eosin staining, x25 views. FI Fiber, GC Giant cell, GP grip, MA Macrophages

The chronic inflammatory reaction observed on the new resorbable mesh was stable with a score of 2 showing a moderate grade 4 weeks up to 20 weeks presenting macrophages and giant cells surrounding the fibers as shown by the Figs. 2, 3. Similar grades of moderate chronic inflammatory reaction (Figs. 2, 3) were observed on TIGR® Matrix Surgical mesh at 4 months in a sheep model [31] and up to 72 weeks on Phasix® mesh in a porcine model [32] with similar density of macrophages and giant cells around the meshes.

#### Evaluation of the new resorbable mesh fixation strength during the tissue integration process

##### Survival, clinical observations

**Fig. 4:**
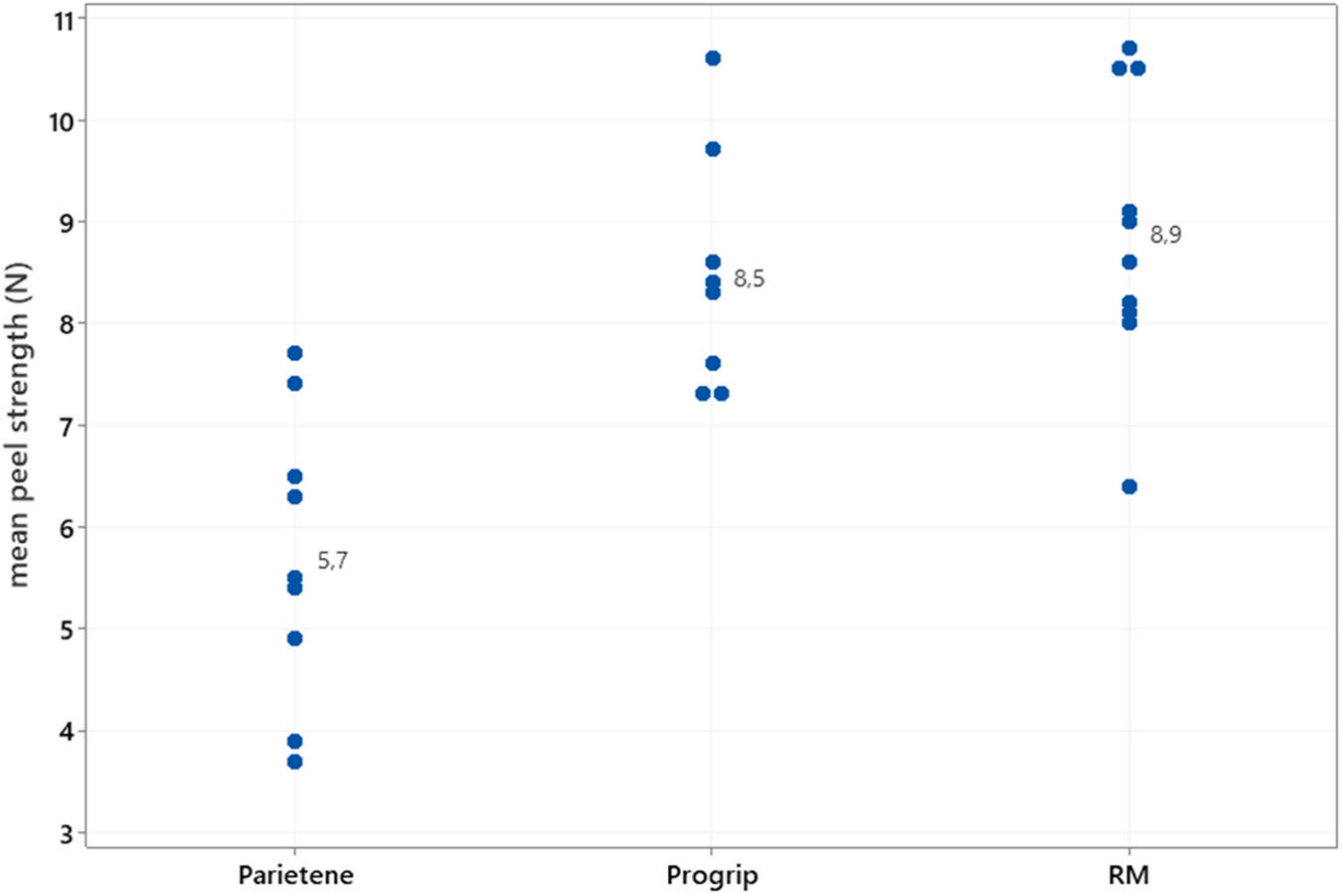
Peeling strengths of explants at 8 weeks, after implantation (Individual values and mean). Parietene: Parietene ™ Flat Sheet Mesh; Progrip: ProGrip™ Self-Gripping Polyester Mesh; RM New Resorbable Mesh

All animals in this study survived until their scheduled euthanasia. Documented postoperative complications were noted. Eight days after the implantation, in two pigs, a slight swelling in the middle part of the scar of the incision closure was observed. No anti-inflammatory treatment was administrated Fig 4. The swelling was resolved 3 days after the first detection. In one pig, a lesion was detected on the left ear of this animal due to the ear tag. The ear tag was removed and blastoestimulina (a local dermal treatment with healing and anti-infective properties) was applied after cleaning the wound. Oral antibiotic treatment was administered for 5 days with Cefadroxil at adose of 20 mg/kg every 24 h.

##### Macroscopic analysis

Eight weeks following implantation, the new resorbable mesh, ProGrip™ Self-Gripping Polyester Mesh and Parietene ™ Flat Sheet Mesh were fully integrated into the abdominal wall. No serous fluid collections, hematomas, herniations, mesh migrations, or areas of tissue necrosis were noted at necropsy.

##### Peeling strength

The peeling strengths of ProGrip™ Self-Gripping Polyester Mesh and the new resorbable mesh were measured at 8.5–8.9 N, on average, 8 weeks after the implantation. Parietene ™ Flat Sheet Mesh without the ProGrip™ technology seems to be less integrated in tissues with observed peeling strengths lower than 6 N on average. The statistical analysis, ANOVA followed by a Fisher’s LSD post-test showed that Parietene ™ Flat Sheet Mesh was significantly different from the new resorbable mesh and ProGrip™ Self-Gripping Polyester Mesh (*p* < 0.05).

The ProGrip™ technology gives a more pronounced 3D configuration to the meshes and a higher volume for tissue integration. The presence of the grips on the entire mesh surface should also allow an overall closer contact between the mesh and the tissues, which should facilitate the integration. It was important to check not only the performance of the new resorbable mesh in an experimental model of hernia, but also to show the quality of the fixation provided by the ProGrip™ technology. Such results are in line with clinical studies that have reported the clinical performance of ProGrip™ meshes in ventral hernia repair [33, 34]. This should support the ease-of-use feature of the new resorbable mesh with facilitated positioning and fixation.

## Conclusion

The hardest requirement does not come from the degradation profile, but from the resistance to mechanical fatigue, i.e., dynamic cyclic loading as it occurs in the clinical environment, for example the repair of defects of the abdominal wall. As indicated in the literature, copolymers of LA and TMC were a key to provide enough flexibility to fight against too early mechanical degradation of PLLA [20–22]. While maintaining slow hydrolytic degradation, the copolymer of TMC and LA specifically designed for the new resorbable mesh provided sufficient mechanical support, at least for the first 20 weeks covering the critical healing period, as shown, both in in vitro settings (cyclic loading test) and in a pig experimental study. Beyond its mechanical properties, the new resorbable mesh was very well tolerated with no adverse effects, as observed in the two animal studies at all implantation times surveyed up to 20 weeks. The mesh also integrates quickly – at least from 4 weeks after implantation – in the abdominal wall with a new connective tissue intertwining closely the mesh as well as the grips which should contribute to a firmer anchorage due to their extension from the mesh and their mushroom shape. This underlines the particularity of the new resorbable mesh with its grips.

The in vitro and the preclinical data of the new resorbable mesh suggest that this new mesh is an adequate candidate for future clinical use.
